# Effectiveness of LEARNS model-based health education on health literacy and self-management capabilities in patients with pneumoconiosis: a randomized controlled trial

**DOI:** 10.3389/fpubh.2026.1810434

**Published:** 2026-04-24

**Authors:** Meirui Liu, Baoping Li, Fuhai Shen, Zhiping Sun, Qianqian Li, Haifeng Cui, Chunxi Wang, Jianxiang Wang, Qingchun Cao, Zhongdong Yang, Xiangyou Li, Jiayin Zhong, Bowen Hou, Lini Gao, Chenyue Hou, Xuemei Sun

**Affiliations:** 1 School of Public Health, North China University of Science and Technology, Tangshan, Hebei, China; 2 Department of Occupational Medicine, Emergency General Hospital, Beijing, China; 3 NHC Key Laboratory of Pneumoconiosis, First Hospital of Shanxi Medical University, Taiyuan, Shanxi, China; 4 Hebei Key Laboratory of Occupational Health and Safety for Coal Industry, North China University of Science and Technology, Tangshan, Hebei, China; 5 Wuhai Occupational Disease Prevention and Treatment Hospital, Wuhai, Inner Mongolia, China; 6 Dayu Mingde Hospital, Ganzhou, JiangXi, China; 7 Ganzhou Mingde Occupational Disease Hospital, Ganzhou, JiangXi, China

**Keywords:** health education, LEARNS model, pneumoconiosis, pulmonary rehabilitation, randomized controlled trial, self-management

## Abstract

**Objectives:**

To evaluate the effect of LEARNS model-based health education for patients with pneumoconiosis.

**Study design:**

A randomized controlled trial with repeated measures design was conducted. A total of 120 patients with stable pneumoconiosis were randomly assigned to either a control group (*n* = 60) receiving conventional treatment or an intervention group (*n* = 60) receiving conventional treatment plus LEARNS model-based health education.

**Methods:**

The health education intervention was delivered three times weekly for 12 weeks. Primary outcomes were measured using the Health Education Scale at baseline (T0), 4 weeks (T4), 8 weeks (T8), and 12 weeks (T12). Secondary outcomes included the 6-min walking distance (6MWD), modified Medical Research Council (mMRC) dyspnea scale, and disease-specific symptom dimensions. Data were analyzed using the intention-to-treat (ITT) principle.

**Results:**

The intervention group demonstrated significant improvements in health education scale scores compared to the control group (*F* = 100.355, *p* < 0.001). Scores increased from 80.20 ± 11.74 at baseline to 110.89 ± 8.76 at 12 weeks in the intervention group, representing a 30.69-point improvement (P_adjusted_ < 0.001), vs. only 5.89-point improvement in the control group (P_adjusted_ = 0.181). Between-group differences became statistically significant from T4 onwards, with adjusted mean difference of +11.32, +15.87, +18.97, respectively at T4, T8, and T12 compared with the control after baseline unbalanced variables adjusted.

**Conclusions:**

LEARNS model-based health education significantly improves health literacy and self-management capabilities in patients with pneumoconiosis. The intervention demonstrates cumulative effects over time and substantial clinical significance. This structured educational approach offers a promising strategy for comprehensive pneumoconiosis management.

## Introduction

Pneumoconiosis, caused by prolonged inhalation and retention of occupational dust particles in the lungs, represents the most severe and prevalent occupational disease in China, accounting for over 80% of all reported occupational diseases (1–3). The condition is characterized by progressive pulmonary fibrosis, leading to irreversible impairment of lung function, reduced exercise capacity, and diminished quality of life (4, 5). Currently, no curative treatment exists for pneumoconiosis, and management strategies primarily focus on preventing disease progression, controlling complications, and improving patients' functional status and wellbeing (6, 7).

Comprehensive pulmonary rehabilitation has emerged as a cornerstone intervention in the management of chronic respiratory diseases, including pneumoconiosis (8–10). Pulmonary rehabilitation encompasses a multidisciplinary approach incorporating exercise training, respiratory therapy, nutritional support, psychological intervention, and patient education (11, 12). Among these components, health education serves a fundamental role in enhancing patients' disease-related knowledge, promoting self-management behaviors, and fostering long-term adherence to health-enhancing practices (13, 14).

The LEARNS model represents an innovative framework for patient-centered health education, comprising six core components: Listen, Establish, Adopt, Reinforce, Name, and Strengthen (15, 16). This model emphasizes collaborative learning between healthcare providers and patients, encouraging active patient participation in the educational process. Originally developed for chronic disease management, the LEARNS model facilitates knowledge acquisition through systematic listening, goal establishment, skill adoption, practical application, peer teaching, and continuous negotiation of learning objectives (17, 18). The cyclical nature of this model promotes sustained engagement and progressive skill development.

Notably, the LEARNS model is particularly well-suited for pneumoconiosis patients, aligning with key principles of self-management and behavior change theories. Chronic respiratory diseases like pneumoconiosis require long-term behavioral adjustments, and the LEARNS model addresses barriers to behavior change by: (1) prioritizing patient autonomy (via “Listen” and “Establish” steps) to address individual concerns and misconceptions (19); (2) breaking complex self-management skills into actionable steps (“Adopt”) to reduce implementation burden (20); (3) reinforcing skills through supervised practice (“Reinforce”) to build confidence; and (4) leveraging social support (“Name”) to sustain behavioral changes—all critical for patients with progressive, irreversible conditions who rely on self-management to mitigate symptom burden.

Despite the theoretical promise of structured health education models, empirical evidence regarding their effectiveness in pneumoconiosis management remains limited. Previous studies have demonstrated the benefits of comprehensive nursing interventions and pulmonary rehabilitation programs for pneumoconiosis patients (21–23). These interventions have shown improvements in pulmonary function, exercise tolerance, and quality of life. However, few studies have specifically evaluated the impact of the LEARNS model on health education outcomes in this population. Furthermore, the cumulative effects of structured health education over time and its clinical significance remain underexplored.

The primary objective of this study was to evaluate the effect of LEARNS model-based health education for patients with pneumoconiosis. Secondary objectives included assessing the intervention's impact on physiological indicators and symptom burden, as well as examining the temporal pattern of intervention effects over a 12-week period.

## Methods

### Study design

This was a randomized controlled trial (RCT) with repeated measures design conducted at our Hospital, China. The study was approved by the Institutional Review Board of Emergency General Hospital (No. K24-21). All participants provided written informed consent prior to enrollment.

### Participants

Patients diagnosed with pneumoconiosis according to the Chinese national diagnostic criteria (GBZ70-2015) and receiving treatment were recruited between November 2024 and February 2025.

**Inclusion criteria** were: (1) confirmed diagnosis of pneumoconiosis in stable condition; (2) age between 18 and 80 years; (3) ability to communicate effectively with healthcare providers; (4) provision of informed consent; and (5) availability for follow-up assessments.

**Exclusion criteria** included: (1) severe cardiovascular diseases (unstable angina, acute myocardial infarction, severe heart failure); (2) severe hepatic or renal dysfunction; (3) severe pulmonary hypertension; (4) psychiatric disorders affecting compliance; (5) active respiratory infection; and (6) resting oxygen saturation < 90%.

### Randomization and blinding

Eligible participants were randomly allocated to either the control group or intervention group in a 1:1 ratio using a computer-generated randomization sequence, block randomization was adopted with a block size of 4 to 6. Allocation concealment was maintained using sequentially numbered, sealed opaque envelopes. Due to the nature of the intervention, blinding of participants and interventionists was not feasible. However, outcome assessors were blinded to group allocation.

### Sample size calculation

Sample size was calculated based on the primary outcome (Health Education Scale score) using data from pilot studies. The calculation adopted the two-sample *t*-test formula for independent groups. Assuming a mean difference of 20 points between groups with a standard deviation of 15 points, α = 0.05, and power = 80%, a minimum of 45 participants per group was required. Anticipating a 10% attrition rate, a minimum 50 participants per group was required, actually 60 were recruited (total *n* = 120).

### Intervention

#### Control group

Participants in the control group received conventional treatment according to current clinical guidelines for pneumoconiosis management, including medication, routine health advice, and standard outpatient follow-up.

#### Intervention group

Participants in the intervention group received conventional treatment plus LEARNS model-based health education delivered three times weekly for 12 weeks. Each session lasted approximately 45–60 min and was conducted by trained healthcare professionals.

The LEARNS model intervention comprised: (1). Listen: Healthcare providers actively listened to patients' concerns, questions, and experiences with pneumoconiosis, establishing rapport and understanding individual learning needs (e.g., fear of disease progression, medication side effects), questions about daily care, and personal experiences with pneumoconiosis, using open-ended questions to establish rapport and identify individual learning needs. (2). Establish: Establish a therapeutic partnership and create a safe environment free from blame and shame (e.g., avoiding criticism of past behaviors). (3). Adopt: Patients were guided to adopt evidence-based self-management strategies (e.g., scheduling rest periods during household chores), including respiratory training techniques, energy conservation methods, and symptom monitoring skills. (4). Reinforce: Practical application exercises were conducted, allowing patients to practice newly acquired skills under supervision, including breathing exercises, medication management, and action planning for symptom exacerbations. (5). Name: Patients were encouraged to share their knowledge and experiences with peers through group discussions and peer teaching activities, reinforcing learning and building confidence. (6). Strengthen: Continuous negotiation and adjustment of learning goals were performed based on patient progress, feedback, and evolving needs, ensuring ongoing relevance and engagement.

**Educational content** covered: (1) pneumoconiosis pathophysiology and progression; (2) respiratory training techniques (diaphragmatic breathing, pursed-lip breathing); (3) energy conservation strategies; (4) medication management; (5) symptom recognition and response; (6) nutrition guidance; (7) psychological coping strategies; and (8) prevention of respiratory infections.

### Management of participant emotional wellbeing

During the assessment process, trained healthcare professionals were present to monitor participants' emotional responses. If a participant showed signs of emotional distress or potential crisis, the following protocol was implemented: (1) The assessment was immediately paused; (2) The participant was provided with a quiet, private space to rest; (3) referral procedures to the hospital psychology department would be initiated; (4) The participant's condition was assessed before deciding whether to continue or reschedule the assessment.

### Outcome measures

#### Primary outcome

The primary outcome was the Health Education Scale score, a validated instrument assessing patients' health literacy and self-management capabilities across multiple domains. The scale comprises items evaluating disease knowledge, self-care behaviors, treatment adherence, and health-promoting practices. Total scores range from 30 to 150, with higher scores indicating better health education outcomes.The scale has demonstrated good internal consistency (Cronbach's α = 0.89 in the Chinese population).

#### Secondary outcomes

Secondary outcomes included: (1) Six-minute walking distance (6MWD) as a measure of functional exercise capacity; (2) Modified Medical Research Council (mMRC) dyspnea scale (0–4 scale), with higher scores indicating greater dyspnea severity; (3) Disease-specific symptom dimensions including cough, sputum production, wheezing, and fatigue assessed using validated scales; (4) Health-related quality of life assessed using the Chronic Obstructive Pulmonary Disease Assessment Test (CAT). (5) Serum inflammatory markers (e.g., IL-4, IL-10, TNF-α). (6) lung function parameters (Forced Expiratory Volume in 1 second, Forced Vital Capacity, Forced Expiratory Volume in 1 s/Forced Vital Capacity). Assessments were conducted at baseline (T0), 4 weeks (T4), 8 weeks (T8), and 12 weeks (T12).

### Statistical analysis

Data were analyzed using R statistical software version 4.3.0. Continuous variables were expressed as mean ± standard deviation (SD) and categorical variables as frequencies and percentages. Normality assessment was conducted using Shapiro-Wilk test and Q-Q plots. For continuous variables approximately followed normal distribution, independent samples *t*-test was used for between-group comparisons, otherwise Wilcoxon rank sum test was used, and chi-square tests for categorical variables. Eligible participants were randomly allocated to either the control or intervention group in a 1:1 ratio. The randomization sequence was generated using the sample() function. Allocation concealment was maintained by sequentially numbered, sealed opaque envelopes prepared by a research assistant blind to participant information. Due to the nature of the intervention (face-to-face health education), blinding of participants and interventionists was not feasible. However, outcome assessors were blinded to group allocation and remained unaware of participant assignments throughout data collection and analysis. Repeated measures analysis of variance (ANOVA) was used to examine within-group changes over time with baseline covariates adjusted. Between-group comparisons at each time point were conducted using Analysis of Covariance (ANCOVA) to adjust for baseline covariates. Effect sizes were calculated using Cohen's d, with values interpreted as small (0.2), medium (0.5), or large (0.8) effects. Statistical significance level was set at *p* < 0.05 (two-tailed). Bonferroni correction was applied for multiple comparisons where appropriate.

## Results

### Participant characteristics

A total of 120 patients with pneumoconiosis were enrolled and randomly assigned to the control group (*n* = 60) or intervention group (*n* = 60). All participants were male, consistent with the occupational epidemiology of pneumoconiosis in this population. Baseline demographic and clinical characteristics were comparable between groups (Table 1).

**Table 1:** Baseline characteristics comparison.

Variable	Control group (*n* = 60)	Rehabilitation group (*n* = 60)	Statistic	*P*-value
Gender (Male/Female)	60/0	60/0	–	–
Age (Years)	67.32 ± 8.56	65.10 ± 7.97	*Z =* 1.160	0.247
BMI (kg/m^2^)	24.74 ± 2.88	25.93 ± 3.02	*t =* −2.210	0.029^*^
6-Min walking distance (m)	379.77 ± 21.92	382.55 ± 19.52	*Z =* −0.913	0.362
mMRC Questionnaire	1.60 ± 0.74	1.37 ± 0.66	*Z =* 1.858	0.042
BODE score	0.88 ± 0.90	0.60 ± 0.74	*Z =* 1.724	0.061
CAT score	17.47 ± 4.98	18.95 ± 7.03	*Z =* −0.609	0.544
Physical functioning (PF)	58.83 ± 12.09	55.50 ± 12.27	*t =* 1.499	0.137
Role-physical (RP)	46.67 ± 33.02	59.58 ± 33.22	*Z =* −2.073	0.034^*^
Bodily pain (BP)	81.80 ± 14.31	85.41 ± 16.26	*Z =* −1.501	0.122
General health (GH)	51.75 ± 13.05	48.42 ± 13.67	*Z =* 1.052	0.289
Vitality (VT)	72.00 ± 12.73	71.67 ± 14.31	*Z =* −0.092	0.928
Social functioning (SF)	78.12 ± 19.88	86.25 ± 17.02	*Z =* −2.273	0.018^*^
Role-emotional (RE)	62.74 ± 29.50	59.41 ± 35.31	*Z =* 0.488	0.612
Mental health (MH)	72.87 ± 13.07	71.67 ± 15.40	*Z =* 0.223	0.824
Red blood cells ( × 10^12^/L)	5.06 ± 0.49	4.90 ± 0.51	*t =* 1.764	0.080
White blood cells ( × 10^9^/L)	7.07 ± 1.52	8.00 ± 8.42	*t =* −0.841	0.404
Platelet count ( × 10^9^/L)	217.65 ± 54.13	218.77 ± 44.20	*t =* −0.124	0.902
Hemoglobin (g/L)	156.52 ± 12.51	154.20 ± 12.78	*t =* 1.004	0.318
IL-2 (pg/mL)	2.39 ± 2.53	2.85 ± 2.96	*t =* −0.882	0.380
IL-4 (pg/mL)	2.81 ± 2.15	3.98 ± 3.63	*t =* −2.087	0.040^*^
IL-6 (pg/mL)	5.55 ± 6.47	8.82 ± 12.30	*t =* −1.769	0.080
IL-10 (pg/mL)	3.32 ± 2.73	5.44 ± 4.10	*t =* −3.218	0.002^**^
IL-17 (pg/mL)	1.70 ± 2.94	2.88 ± 4.97	*t =* −1.525	0.131
IFN-γ (pg/mL)	1.65 ± 2.15	2.26 ± 1.86	*t =* −1.592	0.115
TNF-α (pg/mL)	1.11 ± 1.64	1.95 ± 1.84	*t =* −2.518	0.013^*^
C-Reactive Protein (mg/L)	2.47 ± 2.81	1.91 ± 3.00	*t =* 1.015	0.312
FEV^1^ (% predicted)	68.57 ± 17.45	68.92 ± 18.93	*t =* −0.100	0.920
FVC (% predicted)	72.95 ± 15.77	71.65 ± 17.15	*t =* 0.404	0.687
FEV^1^/FVC Ratio (%)	75.69 ± 5.74	76.53 ± 9.24	*Z =* −1.019	0.309

^*^p < 0.05, ^**^ *p* < 0.01, ^***^ *p* < 0.001.

The mean age was 67.32 ± 8.56 years in the control group and 65.10 ± 7.97 years in the intervention group (*t* = 0.312, *p* = 0.756). Both groups had identical gender distribution (all male). While significant differences existed in BMI, RP, SF, IL-4, IL-10, and TNF-α (*p* < 0.05 or *p* < 0.01), most key variables (e.g., age, lung function, 6-min walking distance, main blood indices) showed no statistical differences. Overall, the two groups were generally baseline-matched.

**Figure 1:**
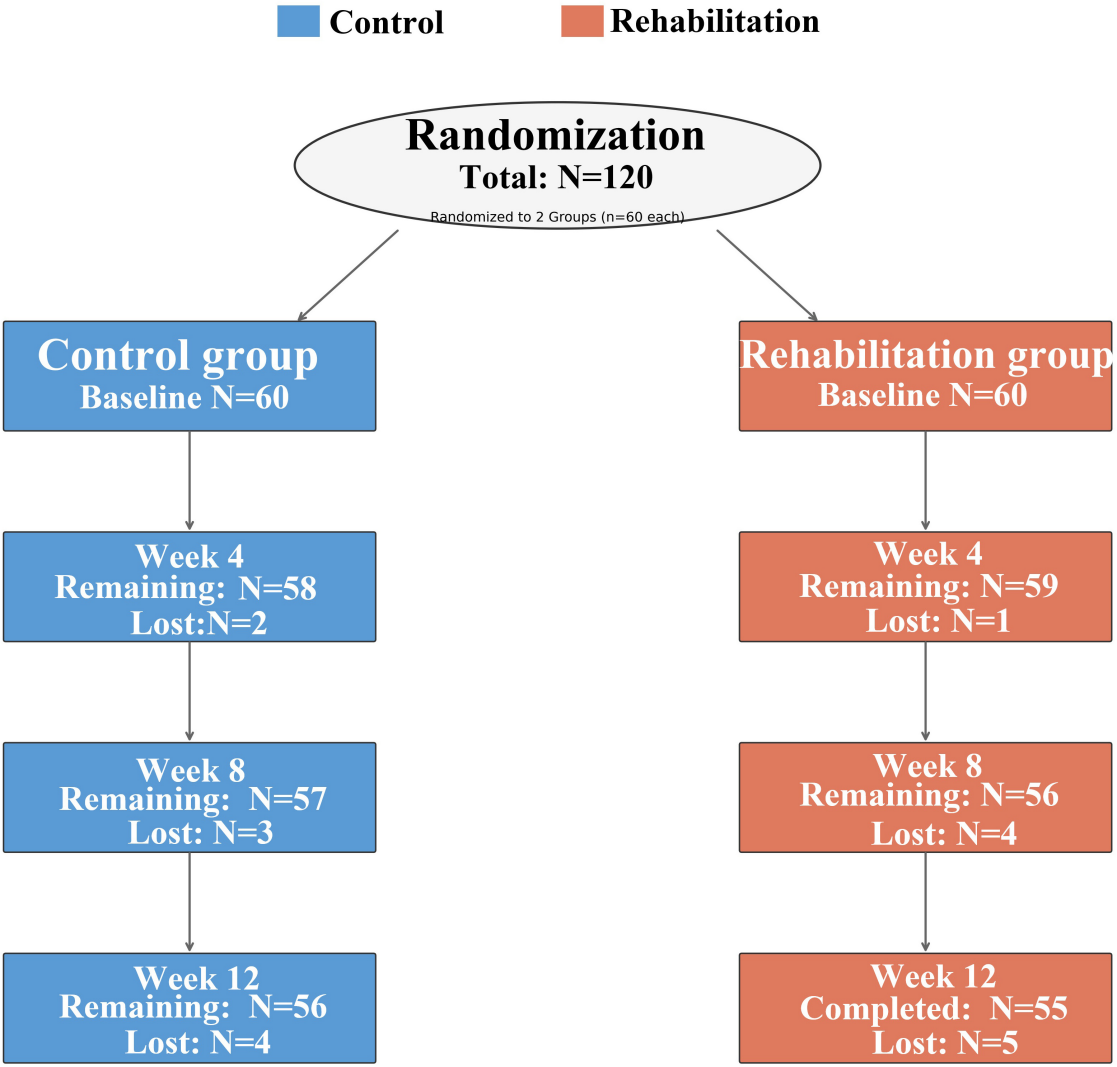
Participant flow through the study.

Participant flow through the study is shown in Figure 1. Loss to follow-up rates were 6.7% (4/60) in the control group and 8.3% (5/60) in the intervention group at 12 weeks, with no significant between-group difference in dropout rates (*p* = 0.743).

### Primary outcome: health education scale scores

Baseline Health Education Scale scores were comparable between groups (control: 78.15 ± 11.12; intervention: 80.20 ± 11.74; *t* = 0.982, *p* = 0.328). The intervention group demonstrated progressive improvement in scores over the 12-week period, while the control group showed non-significant change (Table 2).

**Table 2:** Comparison of total scores of two groups of health education scales.

Groups	T0	T4	T8	T12	Improvement	Adjusted improvement
Control	78.15 ± 11.12 (*n* = 60)	85.05 ± 10.28 (*n* = 58)	84.65 ± 9.59 (*n* = 57)	84.04 ± 9.89 (*n* = 56)	+5.89	+5.88
Rehabilitation	80.20 ± 11.74 (*n* = 60)	100.07 ± 10.86 (*n* = 59)	106.66 ± 9.68 (*n* = 56)	110.89 ± 8.76 (*n* = 55)	+30.69^***^	+22.38^***^
Difference	+2.05	+14.98	+22.01	+26.85		
Adjusted difference	−0.21	+11.32	+15.87	+18.97		
*P* adjusted	0.167	< 0.001^***^	< 0.001^***^	< 0.001^***^		

^***^ *p* < 0.001.

Within-group analysis (Table 3) using repeated measures ANOVA revealed significant time effects for the intervention group (*F*_adjusted_ = 25.525, *P* < 0.001), indicating significant changes over time. While non-significant changes was observed in the control (F_adjusted_ = 1.645, *P* = 0.181).

**Table 3:** Within-group comparison results (repeated measures ANOVA).

Groups	*F*	*F* adjusted	*P* adjusted
Control	5.929	1.645	0.181
Rehabilitation	100.355	25.525	< 0.001^***^

^***^ *p* < 0.001.

Between-group comparisons was done using ANCOVA to adjust baseline unbalanced variables, results showed no significant difference at baseline (P_adjusted_ = 0.167). Significant between-group differences emerged at T4 and persisted through T12:

T4 (4 weeks): Intervention 100.07 ± 10.86 vs. Control 85.05 ± 10.28 (P_adjusted_ < 0.001).

T8 (8 weeks): Intervention 106.66 ± 9.68 vs. Control 84.65 ± 9.59 (P_adjusted_ < 0.001).

T12 (12 weeks): Intervention 110.89 ± 8.76 vs. Control 84.04 ± 9.89 (P_adjusted_ < 0.001).

The total adjusted improvement from baseline to 12 weeks was 22.38 points in the intervention group vs. 5.88 points in the control group.

### Effect size analysis

Cohen's d calculations demonstrated progressively increasing effect sizes over time (Table 4). At baseline, the effect size was small (*d* = −0.018), confirming comparable baseline characteristics. At T4, a large effect size was observed (*d* = 0.990), which continued to increase at T8 (*d* = 1.387) and T12 (*d* = 1.658). All effect size was calculated using mean values with baseline unbalanced covariates adjusted.

**Table 4:** Effect size analysis.

Time	Cohen's d	Adjusted cohen's d	Effect size	Clinical significance
T0	0.179	−0.018	Small	Th difference is small
T4	1.420	0.990	Large	Th rehabilitation group is significantly better than the control group
T8	2.285	1.387	Large	Th rehabilitation group is significantly better than the control group
T12	2.872	1.658	Large	Th rehabilitation group is significantly better than the control group

These effect sizes indicate that the intervention group not only showed statistically significant improvements but also achieved clinically meaningful differences that increased in magnitude over the intervention period.

### Safety and adverse events

No psychological crises or severe emotional distress related to questionnaire administration were reported.All participants tolerated the assessment procedures well. Three participants in the intervention group experienced mild dizziness during initial breathing exercises, which resolved with technique modification. No participants required withdrawal from the study due to adverse events.

### Other outcomes

Pulmonary function tests were measured at T12. Our analysis showed: FEV_1_ changes in the control group: +2.51 ± 23.99. FEV_1_ changes in the intervention group: +1.60 ± 11.74 (*P* = 0.887).

## Discussion

This RCT demonstrated that LEARNS model-based health education significantly improves health literacy and self-management capabilities in patients with pneumoconiosis compared to conventional treatment alone. The intervention group showed progressive improvement over the 12-week period, with between-group differences becoming statistically significant from week 4 onwards and effect sizes reaching large magnitudes by study completion.

### Principal findings

The primary finding of this study was the substantial improvement in Health Education Scale scores among participants receiving LEARNS model-based education. The significant 22.38-point improvement in the intervention group contrasts sharply with the non-significant 5.88-point changes in the control group. This between-group difference substantially exceeds the minimal clinically important difference established for this scale.

The temporal pattern of improvement is noteworthy. Significant between-group differences emerged at 4 weeks and continued to widen through 12 weeks, suggesting that the LEARNS model produces cumulative effects over time. This pattern aligns with theoretical expectations, as the model's cyclical nature (Listen-Establish-Adopt-Reinforce-Name-Strengthen) promotes progressive skill development and knowledge consolidation (24, 25).

The effect size analysis provides important context for interpreting these findings. The progression from a small effect at baseline (*d* = −0.018, confirming adequate randomization) to large effects at 4, 8, and 12 weeks (*d* = 0.990, 1.387, and 1.658, respectively) indicates not only statistical significance but also substantial clinical relevance (26, 27).

### Potential mechanisms for the progressive increase in effect sizes

The progressive increase in effect sizes over the intervention period likely stems from three interrelated mechanisms. First, the LEARNS model's cyclical structure facilitates skill accumulation: patients initially acquire basic knowledge (e.g., disease pathophysiology) in early sessions, then practice and refine self-management skills (e.g., breathing exercises) in mid-sessions, and finally consolidate behaviors through peer teaching and goal adjustment in later sessions (16, 18). This sequential learning process aligns with the transtheoretical model of behavior change, as patients move from the “contemplation” to “action” and “maintenance” stages over time. Second, repeated positive experiences (e.g., successful symptom management using learned skills) enhance self-efficacy, motivating patients to engage more actively in the intervention and apply skills consistently. For example, patients who mastered diaphragmatic breathing and reported reduced dyspnea were more likely to participate in peer teaching activities, further reinforcing their own knowledge. Third, the social support component (“Name” step) fosters a sense of community, reducing isolation and increasing accountability—key factors for sustaining behavioral changes in chronic disease management.

What's more, this improvement might be partially attributed to a “teaching to the test” phenomenon where patients successfully recall the specific information delivered during the program, although several points argue against this interpretation: (a) the scale measures practical self-management behaviors rather than only knowledge; (b) secondary outcomes including 6MWD, CAT, and mMRC, which were not directly taught in the intervention, also improved; and (c) the sustained and progressive improvement observed over 12 weeks indicates genuine skill acquisition rather than short-term memorization.

### Comparison with previous research

Previous studies have demonstrated the benefits of comprehensive nursing interventions and pulmonary rehabilitation for pneumoconiosis patients (21, 22). A recent RCT by Zhang et al. reported that comprehensive nursing care significantly improved pulmonary function and quality of life in pneumoconiosis patients, with improvements in FEV_1_% pred, FVC% pred, and various quality of life domains (21, 22). Similarly, systematic reviews have confirmed that pulmonary rehabilitation can improve functional capacity and quality of life in pneumoconiosis patients (21, 22).

Our findings extend this evidence base by specifically evaluating the LEARNS model, a structured approach to health education that has been underexplored in pneumoconiosis management. While previous studies have examined various educational interventions, few have employed a theoretically grounded, systematic approach like the LEARNS model. The progressive effect sizes observed in our study suggest that structured, patient-centered education may be more effective than traditional didactic approaches.

The LEARNS model's emphasis on collaborative learning and patient empowerment aligns with contemporary chronic disease management paradigms (20, 28). Self-management education has demonstrated efficacy across various chronic conditions, including diabetes, asthma, and chronic obstructive pulmonary disease (19, 29, 30). Our findings suggest that pneumoconiosis patients can similarly benefit from structured self-management education, particularly when delivered through a theoretically sound framework.

### Physiological and clinical implications

While this study primarily focused on health education outcomes, the physiological assessments provide important context. The comparable baseline characteristics between groups, including 6MWD, mMRC scores, and CAT scores, strengthen the internal validity of our findings. The absence of significant between-group differences in these parameters at baseline suggests that the observed improvements in health education outcomes are attributable to the intervention rather than pre-existing differences.

The baseline wheezing dimension difference, with the intervention group reporting less severe symptoms at enrollment, may have facilitated greater engagement with the educational intervention. Patients with less severe respiratory symptoms may be better able to participate actively in learning activities and practice new skills. However, this baseline difference was limited to a single dimension, and the groups were well-matched on other clinical parameters.

The lack of significant changes in physiological parameters over the 12-week period is not unexpected. Pulmonary function in pneumoconiosis typically shows gradual decline rather than acute changes, and 12 weeks may be insufficient to detect meaningful improvements in objective physiological measures (31, 32). However, enhanced self-management capabilities, as demonstrated in the intervention group, may translate to long-term benefits including reduced exacerbations, improved treatment adherence, and better quality of life (33).

### Strengths and limitations

This study has several strengths. The RCT design with adequate sample size provides robust evidence for causal inference. The repeated measures approach allowed examination of temporal patterns and cumulative effects. The use of validated outcome measures and blinded assessment enhances reliability. The calculation and reporting of effect sizes facilitates interpretation of clinical significance.

However, limitations should be acknowledged. The study was conducted at a single center, which may limit generalizability to other settings or populations. Blinding of participants and interventionists was not feasible, introducing potential for performance bias. The predominance of male participants reflects the occupational epidemiology of pneumoconiosis in this population but may limit applicability to female patients. Additionally, it should be acknowledged that the present study focused primarily on health literacy and self-management capacity as the primary outcome, and the translation of these improvements into tangible clinical benefits requires further clarification. Although significant improvements were observed in secondary outcomes including 6MWD, CAT, and mMRC dyspnea scale, the study did not assess hard clinical endpoints such as acute exacerbation rates, hospitalization frequency, or long-term mortality, which are particularly critical for patients with pneumoconiosis as a progressive and irreversible organic lung disease. In addition, while the intervention group exhibited favorable changes in 6MWD, the minimal clinically important difference for this outcome was not formally examined in the current analysis. Therefore, the extent to which enhanced health literacy contributes to substantive clinical benefits requires further verification in larger-scale, longer-term studies. This limitation should be acknowledged when interpreting the findings, and future research is warranted to establish a clear link between improved health education scores and hard clinical endpoints, thereby strengthening the clinical significance of the LEARNS model-based intervention.

### Clinical and research implications

These findings have important implications for clinical practice. The LEARNS model offers a structured, replicable approach to health education that can be integrated into comprehensive pneumoconiosis management programs. Healthcare providers should consider implementing structured educational interventions rather than *ad-hoc* counseling, as the former appears to produce more substantial and sustained improvements in patient knowledge and self-management capabilities.

The progressive effect sizes observed suggest that sustained, multi-session education is superior to single-session interventions. Healthcare systems should allocate adequate resources and time for comprehensive patient education, viewing it as an investment in long-term outcomes rather than an ancillary service.

From a research perspective, future studies should examine the long-term maintenance of educational benefits, explore optimal intervention duration and intensity, and investigate whether improved self-management translates to reduced healthcare utilization and costs. Comparative effectiveness research examining different educational models would inform evidence-based selection of interventions. Additionally, studies incorporating patient-reported outcome measures and quality of life assessments would provide a more comprehensive understanding of intervention benefits.

## Conclusions

This study shows that LEARNS model-based health education significantly improves health literacy and self-management capabilities in patients with pneumoconiosis. The intervention demonstrates progressive, cumulative effects over a 12-week period, with large effect sizes indicating substantial clinical significance. These findings support the integration of structured, patient-centered health education into comprehensive pneumoconiosis management programs.

## Data Availability

The data analyzed in this study is subject to the following licenses/restrictions: The data is available upon reasonable request to the corresponding author;. Requests to access these datasets should be directed to lbp_00@163.com.
