# Supplementary material for: Effectiveness of LEARNS model-based health education on health literacy and self-management capabilities in patients with pneumoconiosis: a randomized controlled trial
Source: Front Public Health. 2026 Apr 24;14:1810434. doi: 10.3389/fpubh.2026.1810434 (PMC13153088; doi:10.3389/fpubh.2026.1810434)
Supplement: Supplementary file 1 [file Data_Sheet_1.docx]

Appendix Health Education Evaluation Form

| **No.** | **Items** | **Never** | **Rarely** | **Sometimes** | **Often** | **Always** |
| --- | --- | --- | --- | --- | --- | --- |
| 1 | Perform breathing exercises when not experiencing shortness of breath | □ | □ | □ | □ | □ |
| 2 | Take medication as prescribed when feeling shortness of breath | □ | □ | □ | □ | □ |
| 3 | Comply with smoking cessation advice | □ | □ | □ | □ | □ |
| 4 | Develop and maintain good sleep habits | □ | □ | □ | □ | □ |
| 5 | Develop and maintain good personal hygiene habits | □ | □ | □ | □ | □ |
| 6 | Take active whole‑body exercise and adjust intensity appropriately according to physical condition | □ | □ | □ | □ | □ |
| 7 | Increase intake of high‑quality protein | □ | □ | □ | □ | □ |
| 8 | Reduce intake of high‑calorie foods | □ | □ | □ | □ | □ |
| 9 | Increase intake of fresh fruits and vegetables | □ | □ | □ | □ | □ |
| 10 | Eat small, frequent meals and maintain good eating habits | □ | □ | □ | □ | □ |
| 11 | Adjust clothing appropriately with weather changes to prevent colds | □ | □ | □ | □ | □ |
| 12 | Use heating facilities to keep warm in winter | □ | □ | □ | □ | □ |
| 13 | Wear enough clothes to prevent wind and cold in cold weather | □ | □ | □ | □ | □ |
| 14 | Take precautions against respiratory tract infections | □ | □ | □ | □ | □ |
| 15 | Learn methods of coughing and expectorating sputum | □ | □ | □ | □ | □ |
| 16 | Avoid overexertion | □ | □ | □ | □ | □ |
| 17 | Regulate emotions through exercise | □ | □ | □ | □ | □ |
| 18 | Conduct self‑psychological counseling to overcome fear | □ | □ | □ | □ | □ |
| 19 | Exchange psychological feelings with other similar patients | □ | □ | □ | □ | □ |
| 20 | Shift attention to other activities when in a bad mood | □ | □ | □ | □ | □ |
| 21 | Maintain a good mood and a positive, optimistic attitude | □ | □ | □ | □ | □ |
| 22 | Access disease‑related knowledge through various ways | □ | □ | □ | □ | □ |
| 23 | Participate in health education activities and learn relevant knowledge | □ | □ | □ | □ | □ |
| 24 | Exchange psychological feelings with other pneumoconiosis patients | □ | □ | □ | □ | □ |
| 25 | Have confidence in following the doctor’s instructions | □ | □ | □ | □ | □ |
| 26 | Maintain an optimistic attitude toward pneumoconiosis | □ | □ | □ | □ | □ |
| 27 | Popularize health education knowledge among other pneumoconiosis patients | □ | □ | □ | □ | □ |
| 28 | Consult medical staff about pneumoconiosis‑related knowledge | □ | □ | □ | □ | □ |
| 29 | Avoid public places during influenza seasons | □ | □ | □ | □ | □ |
| 30 | Avoid raw, cold, hard or spicy irritating foods | □ | □ | □ | □ | □ |

Note: Never=1; Rarely=2; Sometimes=3; Often=4; Always=5
